# Association between semen microbiome disorder and sperm DNA damage

**DOI:** 10.1128/spectrum.00759-24

**Published:** 2024-06-20

**Authors:** Junxian He, Menghui Ma, Zhenhan Xu, Jintao Guo, Haicheng Chen, Xing Yang, Peigen Chen, Guihua Liu

**Affiliations:** 1 Reproductive Medicine Center, The Sixth Affiliated Hospital, Sun Yat-sen University, Guangzhou, China; 2 GuangDong Engineering Technology Research Center of Fertility Preservation, Guangzhou, China; 3 Biomedical Innovation Center, The Sixth Affiliated Hospital, Sun Yat-sen University, Guangzhou, China; 4 Key Laboratory of Human Microbiome and Chronic Diseases (Sun Yat-sen University), Ministry of Education, Guangzhou, China; Children's National Hospital, Washington, DC, USA

**Keywords:** microbiota, RNA, ribosomal, 16S, metabolomics

## Abstract

DNA fragmentation index (DFI), a new biomarker to diagnose male infertility, is closely associated with poor reproductive outcomes. Previous research reported that seminal microbiome correlated with sperm DNA integrity, suggesting that the microbiome may be one of the causes of DNA damage in sperm. However, it has not been elucidated how the microbiota exerts their effects. Here, we used a combination of 16S rRNA sequencing and untargeted metabolomics techniques to investigate the role of microbiota in high sperm DNA fragmentation index (HDFI). We report that increased specific microbial profiles contribute to high sperm DNA fragmentation, thus implicating the seminal microbiome as a new therapeutic target for HDFI patients. Additionally, we found that the amount of *Lactobacillus* species was altered: *Lactobacillus iners* was enriched in HDFI patients, shedding light on the potential influence of *L. iners* on male reproductive health. Finally, we also identified enrichment of the acetyl-CoA fermentation to butanoate II and purine nucleobase degradation I in the high sperm DNA fragmentation samples, suggesting that butanoate may be the target metabolite of sperm DNA damage. These findings provide valuable insights into the complex interplay between microbiota and sperm quality in HDFI patients, laying the foundation for further research and potential clinical interventions.

**IMPORTANCE:**

The DNA fragmentation index (DFI) is a measure of sperm DNA fragmentation. Because high sperm DNA fragmentation index (HDFI) has been strongly associated with adverse reproductive outcomes, this has been linked to the seminal microbiome. Because the number of current treatments for HDFI is limited and most of them have no clear efficacy, it is critical to understand how semen microbiome exerts their effects on sperm DNA. Here, we evaluated the semen microbiome and its metabolites in patients with high and low sperm DNA fragmentation. We found that increased specific microbial profiles contribute to high sperm DNA fragmentation. In particular, *Lactobacillus iners* was uniquely correlated with high sperm DNA fragmentation. Additionally, butanoate may be the target metabolite produced by the microbiome to damage sperm DNA. Our findings support the interaction between semen microbiome and sperm DNA damage and suggest that seminal microbiome should be a new therapeutic target for HDFI patients.

## INTRODUCTION

Recent meta-analyses of semen parameters from 1973 to 2019 indicate a significant global decline in sperm concentration (SC) and total sperm count (TSC), with the rate of reduction accelerating (1). Inferior sperm quality has been strongly associated with adverse reproductive outcomes (2). Nonetheless, the predictive capacity of routine semen analysis for assessing male fecundity is constrained (3). The DNA fragmentation index (DFI), a measure of sperm DNA fragmentation, has gained clinical relevance (4). Factors, such as reactive oxygen species (ROS) (5) and inflammatory agents (6), have been identified as contributors to sperm DNA damage. High levels of sperm DNA fragmentation are associated with reduced fertilization rates, poor embryo quality, lower pregnancy success, and increased miscarriage rates (7).

Current treatments for high DNA fragmentation index (HDFI) include antioxidant therapy, infection management, and lifestyle changes (8), but these approaches often prove inadequate, especially in cases of idiopathic HDFI (9). This has led to an increased reliance on assisted reproductive technologies (ARTs) (5), yet the success rates vary, underscoring the need for alternative treatment strategies.

The human microbiome, encompassing the collective genetic material of microbiota in various body niches, plays a vital role in physiological homeostasis (10). Imbalances in the microbiome can lead to various pathologies (11). Recent research has begun to explore the connection between the semen microbiome and semen quality (12). Notably, a study by Sergio et al. using 16S rRNA gene sequencing revealed a negative correlation between certain bacteria and sperm DNA fragmentation in a Western Mediterranean cohort (13). Thus, our study aims to further investigate the role of the semen microbiome in HDFI using both 16S rRNA and metabolome sequencing. We hypothesize that the seminal microbiota could significantly influence sperm DNA integrity, potentially offering new avenues for HDFI treatment.

## MATERIALS AND METHODS

### Study population and tissue sample collection

All semen samples were taken during the participants’ abstinence period of 3–7 days and were left to liquefy for 30 min. Before collection, the subjects were instructed on the proper procedures to minimize the risk of contamination. The subjects washed their hands with soap two or three times, and the penis, including the glans and coronal sulcus, was cleaned with warm soapy water and then swabbed with 75% alcohol two or three times. Semen was ejaculated directly into a sterile glass receptacle, ensuring no contact with the inner walls to maintain sterility. Freshly collected semen was used for routine clinical tests, Gram staining, and microscopy. The reference for semen analysis is the sixth edition of the WHO laboratory manual for the examination and processing of human semen. Any remaining semen samples were transferred to sterile freezing tubes and placed in liquid nitrogen for at least 15 min, before being transferred to −80°C freezer for storage until the next sample-processing session.

Our inclusion criteria were the following: (i) DFI >30% or <15%; (ii) no family history of genetic disease; (iii) no history of genetic disease, systemic disease, known diseases such as varicocele that can affect DFI, or long-term exposure to radiation and toxic substances; (iv) no history of sexually transmitted diseases in the past 3 months; (v) no systemic corticosteroid, prescription antibiotics, immunosuppressive drugs, systemic corticosteroid use, or cancer chemotherapy in the past 3 months; (vi) no evidence of genitourinary infection.

### Sperm chromatin structure assay

In accordance with the protocols delineated by the manufacturer, the DNA fragmentation index (DFI) was assessed using acridine orange dye supplied by Zhuhai Anda Biological Engineering Co., LTD. The fluorescence patterns were captured utilizing a flow cytometer, specifically the DxPAthena B4-R2 model from Cytek Biosciences. The calculation of the DFI was then achieved by determining the ratio of red to total fluorescence intensity (the sum of red and green fluorescence intensities) (14, 15).

### Cross-incubation experiment

Semen specimens were procured from 15 individuals, stratified into three distinct groups: (i) sperm donor group (*n* = 5); (ii) high DNA fragmentation index (HDFI) group (*n* = 5); and (iii) low DNA fragmentation index (LDFI) group (*n* = 5). A segment of each specimen was immediately used to ascertain the DNA fragmentation index (DFI-before).

For HDFI and LDFI groups, the specimens were centrifuged twice at 1800 rpm for 10 min at 37°C, and seminal plasma was segregated and stored in discrete tubes. For the sperm group, a refined density gradient centrifugation technique was utilized to segregate spermatozoa from seminal plasma to augment sperm quality. Specifically, gradient liquid was gently introduced to the upper layer of semen, followed by centrifugation at 300 × *g* (1,800 rpm) for 10 min at 37°C. The supernatant was subsequently discarded, and the sedimented material was transferred into phosphate-buffered saline (PBS; Servicebio, China), followed by another centrifugation at 300×*g* (1,800 rpm) for 5 min. This procedure was repeated twice to ensure the complete removal of residual gradient liquid, culminating in the resuspension of the sperm pellet in PBS. The treated semen specimens from the sperm group were then divided into three equivalent parts and added to either to the seminal plasma of the HDFI or LDFI group or to an identical volume of PBS.

Preliminary evaluations of the DNA fragmentation index (DFI-T0) were executed on each semen specimen. Thereafter, the specimens were subjected to a 24-h incubation at 37°C. The specimens added in PBS were designated as DFI-T24, whereas those in the seminal plasma of the HDFI and LDFI groups were labeled as HDFI-T24 and LDFI-T24, respectively.

### Microbial total DNA extraction and sequencing

In this study, seven negative controls were meticulously implemented to minimize the risk of contamination, including potential sources: glass receptacle, both unopened and opened freezing tubes, ambient air, tabletop surfaces, and reagents, aligning with recommendations from a previous study (16). Then, total genomic DNA was extracted using MagPure Soil DNA LQ Kit (Magan) following the manufacturer’s instructions. NanoDrop 2000 (Thermo Fisher Scientific, USA) and agarose gel electrophoresis were used to detect the concentration and purity of microbial DNA, and the extracted DNA was stored at −20°C.

For the amplification of bacterial 16S rRNA genes, PCR was conducted using barcoded primers specific to the sequence, in conjunction with Takara Ex Taq (Takara), utilizing the previously extracted DNA as a template. The bacterial diversity analysis emphasized the amplification of the V3–V4 variable regions of the 16S rRNA genes, employing universal primers 343F (5′-TACGGRAGGCAGCAG-3′) and 798R (5′-AGGGTATCTAATCCT-3′) (17). After secondary purification, the final amplicon was quantified using Qubit dsDNA Assay Kit (Thermo Fisher Scientific, USA). Sequencing of the constructed libraries was performed on an Illumina NovaSeq 6000 with 250-bp paired-end reads (Illumina Inc., San Diego, CA; OE Biotech Company; Shanghai, China).

### 16S rRNA data analysis

The raw reads underwent a series of pre-processing steps. First, Cutadapt (18) software was used to detect and cut off the adapter. Following trimming, the paired-end reads underwent a series of operations, including filtering low-quality sequences, denoising, merging, and identifying and removing chimera reads. This was achieved through the application of DADA2 (19) with the default parameters of QIIME 2 (2020.11) (20). Subsequently, the software generated representative reads and an abundance table for amplicon sequence variants (ASVs). The representative read for each ASV was selected using the QIIME2 package. To enhance taxonomic classification, all representative reads were annotated and subjected to a BLAST search against the Silva database (Version 138) (21) using q2-feature-classifier with default parameters. To enhance the differentiation of genuine low-biomass signals from contamination and noise, efforts should be directed toward enhancing the alignment of experimental and computational pipelines. We utilized the SCRuB (22) software to eliminate contaminating reads identified through negative control samples.

### Community diversity analysis

The α and β diversity analyses were analyzed using the QIIME2 software. The α-diversity is assessed using the Shannon index. The unweighted Unifrac distance matrix performed by R package was used for non-metric multidimensional scaling (NMDS) to estimate the β diversity. Then, the R package was used to analyze the significant differences between different groups using the ANOSIM statistical test.

### Microbial composition difference analysis

The taxonomy abundance spectrum was compared using the linear discriminant analysis effect size (LEfSe) method. To investigate the potential utility of microbiota in disease diagnosis and prediction, we utilized a machine learning approach, specifically random forest, to construct classification models based on the level of DFI. First, we calculated feature importance scores using the random forest algorithm, specifically the mean decrease accuracy, which indicates the contribution of each feature to the accuracy of the model. Subsequently, we conducted 10 trials of 10-fold cross-validation using random forest to identify optimal biomarkers. The cutoff point for selecting optimal biomarkers was determined based on the mean of the minimum cross-validation error. Random forest analysis was conducted using R software. The optimal biomarker sets were selected based on the cutoff point in the cross-validation error curve, defined as the minimum cross-validation error. Redundancy analysis (RDA) was performed in R (http://cran.r-project.org/) using the vegan package with normalized OTU abundance and environmental chemical data. Redundancy analysis (RDA) bi-plot shows the correlation between the sequence abundance of microbiota communities and the environmental variables.

### Non-targeted metabolic profiling

Semen samples were subjected to metabolomics analysis. Non-targeted metabolic profiling procedure was followed as described previously (23). Semen samples were thawed at room temperature and mixed with L-2-chlorophenylalanine as an internal standard. After vortexing, an ice-cold mixture of methanol and acetonitrile was added, followed by ultrasonication and storage at −20°C. The supernatant was collected after centrifugation, dried, and reconstituted with a mixture of methanol and water. After further vortexing and ultrasonication, the samples were stored at −20°C and centrifuged, and the supernatants were filtered and transferred to LC vials for storage at −80°C until LC–MS analysis. Quality control (QC) samples were prepared by pooling aliquots of all samples. Metabolomic analysis was conducted using an ACQUITY UPLC I-Class plus (Waters Corporation, Milford, USA) coupled with a Q-Exactive mass spectrometer. A gradient elution system of water and acetonitrile was used for separation on an ACQUITY UPLC HSS T3 column. The mass range was from *m*/*z* 100 to 1,000, with a resolution of 70,000 for full MS scans and 17,500 for HCD MS/MS scans. The mass spectrometer settings included spray voltages, gas flow rates, and temperature control for optimal performance.

### Non-targeted metabolic data analysis

The matrix was imported into R software (v.4.2.2) for conducting principal component analysis (PCA) to observe the overall distribution among the samples and assess the stability of the entire analysis process. Orthogonal partial least-squares discriminant analysis (OPLS-DA) and partial least-squares discriminant analysis (PLS-DA) were utilized to identify metabolites that exhibit differences between groups. To mitigate overfitting, sevenfold cross-validation and 200 response permutation testing (RPT) were utilized to assess the model’s quality. Variable importance of projection (VIP) values obtained from the OPLS-DA model were used to rank the overall contribution of each variable to group discrimination. Subsequently, a two-tailed Student’s *t*-test was applied to validate the significance of differences in metabolites between groups. Differential metabolites were selected based on VIP values >1.0 and *P *< 0.05.

Finally, the distinct metabolites were annotated using the metabolic pathways available in the KEGG database (https://www.kegg.jp/kegg/pathway.html) to determine the pathways associated with these metabolites. Pathway enrichment analysis was conducted using the Python software package scipy.stats (https://docs.scipy.org/doc/scipy/), with the most relevant biological pathway related to the level of DFI identified through Fisher’s exact test.

## RESULTS

### Subject recruitment and semen collection

**Fig 1:**
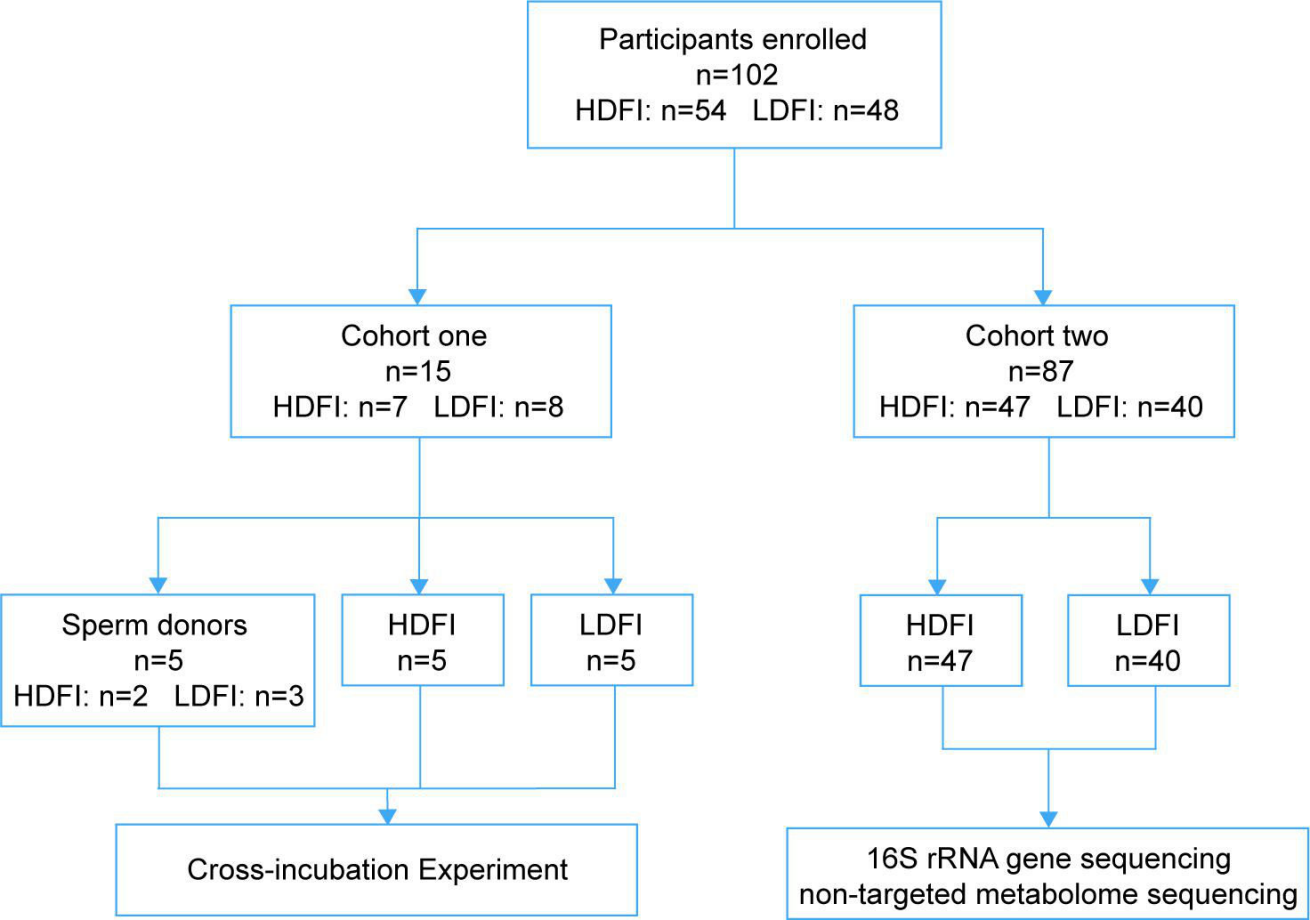
Participants enrolled in the study.

In this study, 102 participants were enrolled, including 54 men with HDFI (DFI > 30%) and 48 men with LDFI (DFI < 15%). The enrolled participants were divided into two cohorts (Fig. 1): for cohort one, cross-incubation experiments were performed on 10 participants, with another five participants who served as sperm donors; for cohort two, 16S rRNA sequencing alongside non-targeted metabolic profiling was conducted on semen samples from 87 participants, comprising 40 with LDFI and 47 with HDFI. Clinical characteristics across the HDFI and LDFI groups were carefully matched (Tables 1 and 2).

**TABLE 1:** Clinical parameters of cohort one participants^*a*^

Parameters	HDFI group (*n* = 5)	LDFI group (*n* = 5)	*P*
Age (years)	35.00 ± 4.000	29.80 ± 3.633	0.0636
DFI (%)	44.60 ± 12.09	6.690 ± 2.264	0.0001
pH	7.440 ± 0.0894	7.440 ± 0.0894	>0.9999
Sperm concentration	46.62 ± 20.71	65.93 ± 49.75	0.4462
Total number of spermatozoa (×10^6^)	104.1 ± 42.48	278.8 ± 259.8	0.176
Progressive motility sperm (10^6^/mL)	28.60 ± 2.302	68.00 ± 14.58	0.0003
Non-progressive sperm (10^6^/mL)	9.800 ± 2.168	12.40 ± 3.912	0.2298
Immotile sperm (10^6^/mL)	61.60 ± 3.209	19.60 ± 15.82	0.004

^*a*^ DFI, DNA fragmentation index; HDFI, high sperm DNA fragmentation index; LDFI, low sperm DNA fragmentation index.

**TABLE 2:** Clinical parameters of cohort two participants^*a*^

Parameters	HDFI group (*n* = 47)	LDFI group (*n* = 40)	*P*
Age (years)	39.72 ± 6.156	38.88 ± 4.195	0.4628
DFI (%)	39.89 ± 9.993	9.649 ± 3.535	<0.0001
pH	7.406 ± 0.136	7.433 ± 0.135	0.3721
Sperm concentration	66.8 ± 57.21	80.53 ± 58.69	0.2739
Total number of spermatozoa (×10^6^)	249.2 ± 202.3	239.4 ± 128.7	0.7928
Progressive motility sperm (10^6^/mL)	17.27 ± 21.17	41.15 ± 33.07	0.0001
Non-progressive sperm (10^6^ /mL)	8.979 ± 13.59	16.51 ± 19.34	0.0365
Immotile sperm (10^6^/mL)	42.70 ± 35.11	22.87 ± 14.83	0.0013

^*a*^ DFI, DNA fragmentation index; HDFI, high sperm DNA fragmentation index; LDFI, low sperm DNA fragmentation index.

### Effect of seminal plasma composition on DFI

**Fig 2:**
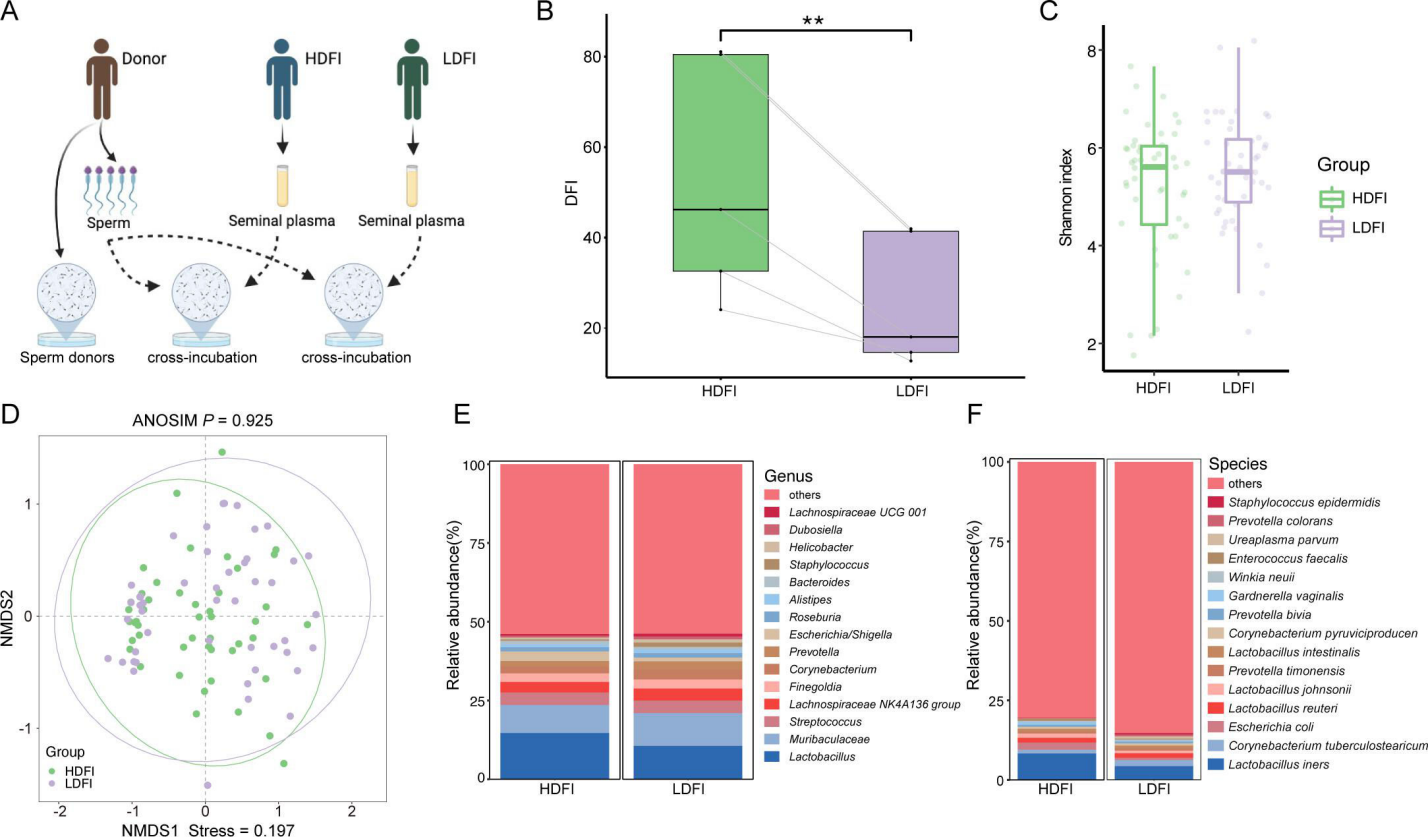
Overview of cross-incubation and composition of the seminal microbiota. (**A**) The experimental workflow for cross-incubation. (**B**) Boxplots illustrating the level of DFI after cross-incubation with seminal plasma from HDFI or LDFI groups. (**C**) Boxplots comparing the Shannon index α-diversity of microbial communities in HDFI and LDFI groups. (**D**) Principal-component analysis plots of β-diversity in the two groups. Stacked plot of genus composition of HDFI and LDFI groups at the genus level (**E**) and species level (**F**).

To investigate the impact of seminal plasma composition on DFI, cross-incubation experiments were conducted (Fig. 2A). Notably, an increase in DFI was observed following incubation with plasma from high DFI patients, suggesting that microbiota-derived compositions within seminal plasma could modify the semen environment (Fig. 2B; Table S1).

### Seminal microbiota composition

Initial analyses compared overall microbiome community compositions between the HDFI and LDFI groups. However, no significant differences were found in either α-diversity (Shannon index) or β-diversity (ANOSIM: *P*＞0.05) (Fig. 2C and D). The subsequent analysis focused on identifying specific taxa with significant differences in abundance between the two groups, revealing notable changes in genera, including *Escherichia–Shigella* and *Lactobacillus* spp., with a significant increase in *L. iners* within the HDFI group (Fig. 2E and F).

### Relationship between seminal microbiota and level of DFI

**Fig 3:**
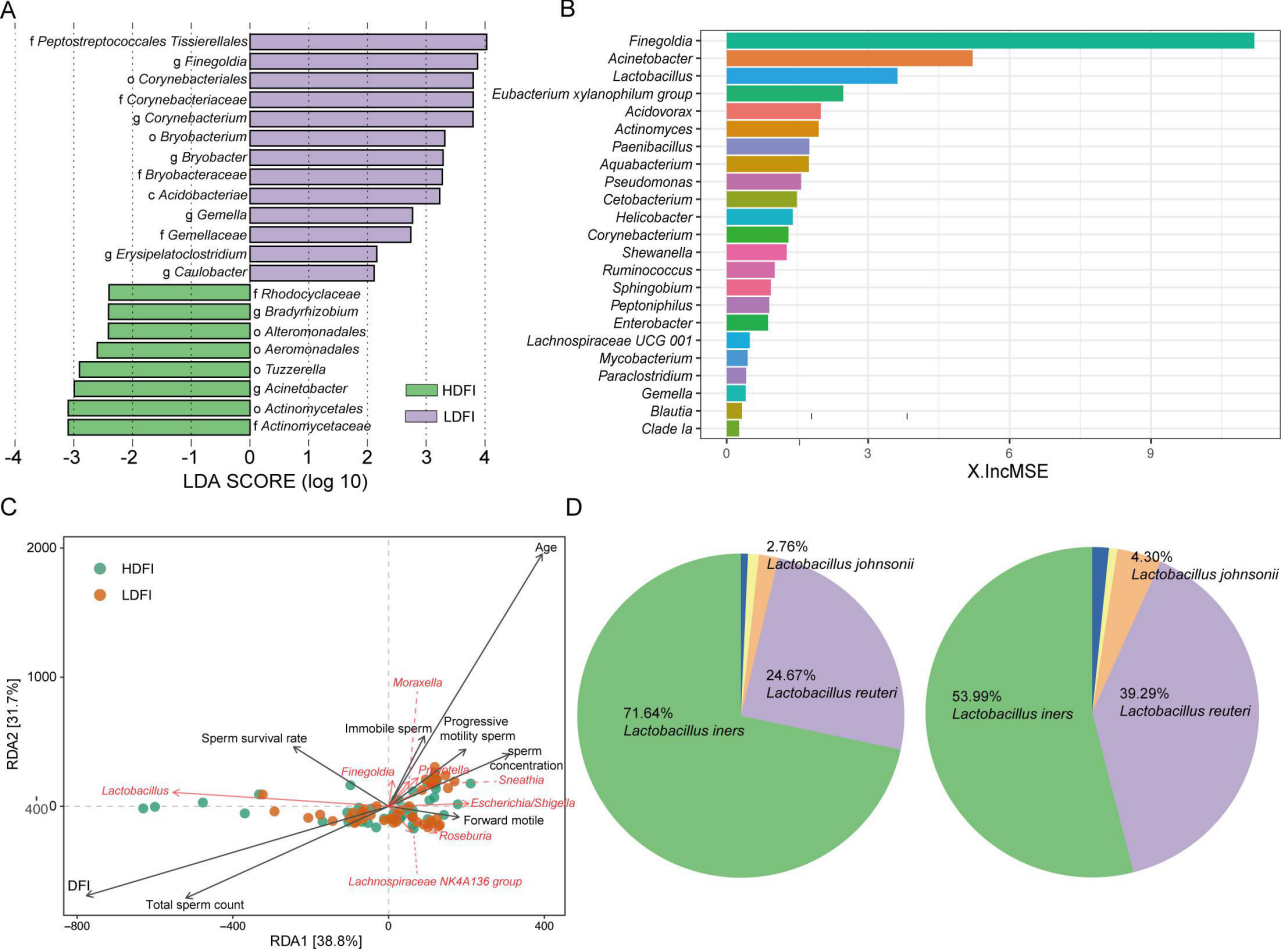
Bioinformatic analysis of 16S rRNA gene sequencing data. (**A**) Linear discrimination analysis (LDA) effect size (LEfSe) analysis identified the microbes whose abundances significantly differed between the HDFI and LDFI groups. (**B**) Model candidates for disease discrimination were established using random forest model analysis. (**C**) Biplot of redundancy analysis (RDA) of the microbiota composition responding to the level of DFI. (**D**) Pie chart showing distribution proportions of different *Lactobacillus* species.

The linear discriminant analysis effect size (LEfSe) method identified differential bacteria at the genus level, highlighting enrichments of *Peptostreptococcales–Tissierellales, Finegoldia* spp.*,* and *Corynebacterium* spp. in the LDFI group and *Acinetobacter* spp. in the HDFI group (Fig. 3A). Random forest analysis further pinpointed *Finegoldia* spp., *Acinetobacter* spp., and *Lactobacillus* spp. as significant contributors to the HDFI and LDFI group classifications (Fig. 3B). Through redundancy analysis, we identified key *Lactobacillus* spp. that correlated with the level of DFI (Fig. 3C). Subsequently, we compared the differences in various *Lactobacillus* species between HDFI and LDFI groups and found significant alterations in the distribution proportions of different *Lactobacillus* species, particularly *L. iners* (Fig. 3D). Additionally, *L. iners* was the most prevalent species in the HDFI group. Together, our results showed that microbial community structures were different between HDFI and LDFI groups.

### Differential microbiota and KEGG pathways

**Fig 4:**
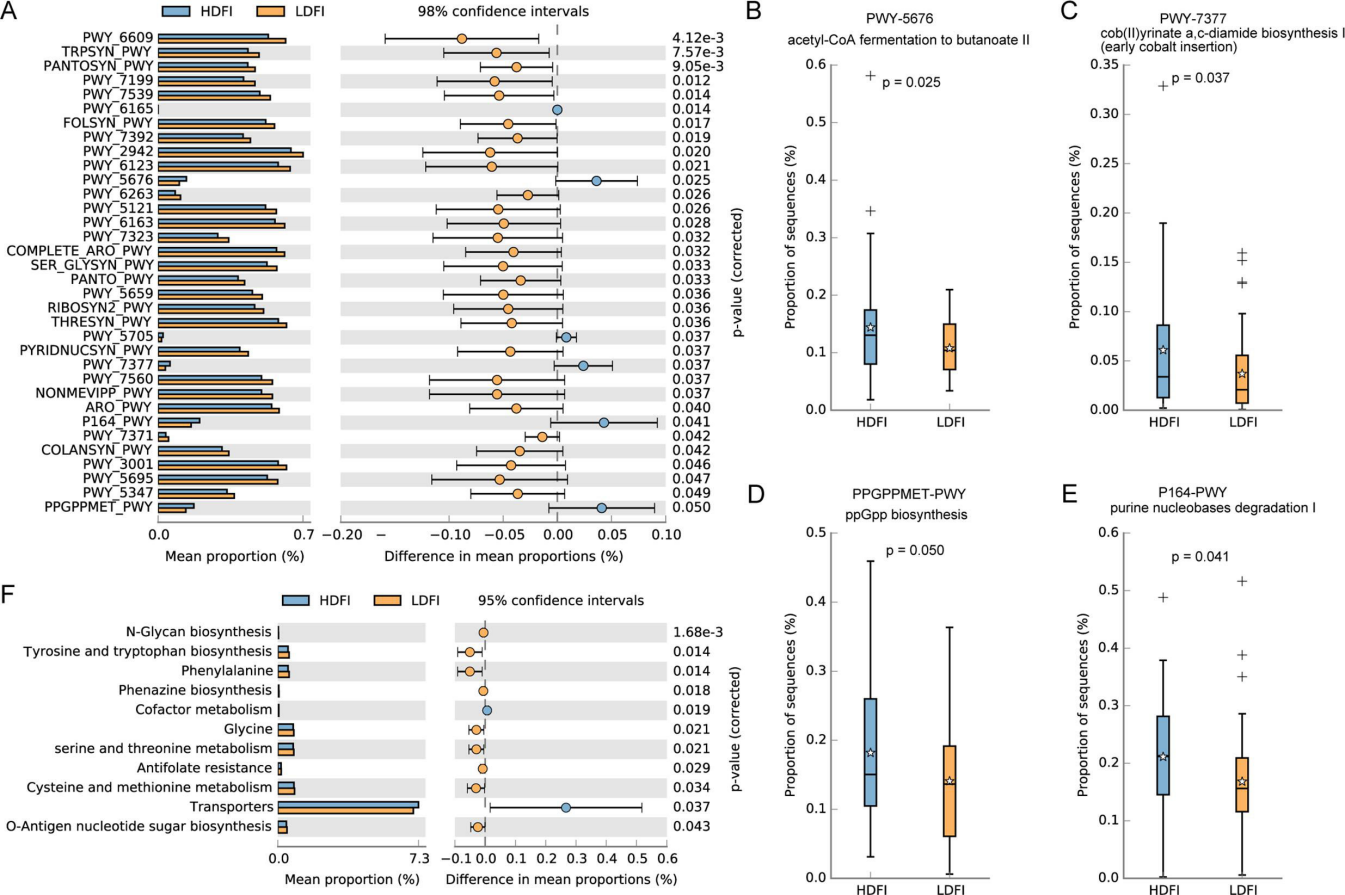
Functional alterations of the microbiota. Difference analyses of KEGG orthology (**A–E**) and metabolic pathways from all domains of life (metacyc) (**F**).

Functional analysis of the differential seminal microbiota revealed enrichment in 34 metabolic pathways (Fig. 4A), with the HDFI group showing significantly higher activity in pathways related to acetyl-CoA fermentation to butanoate II, cob(II)yrinate a,c-diamide biosynthesis I, ppGpp biosynthesis, and purine nucleobase degradation I (Fig. 4B through E). Differential microbiota in the LDFI group exhibited a pronounced enrichment in amino acid metabolism (Fig. 4F).

### Semen microbiota alters sperm DFI by influencing metabolite composition

**Fig 5:**
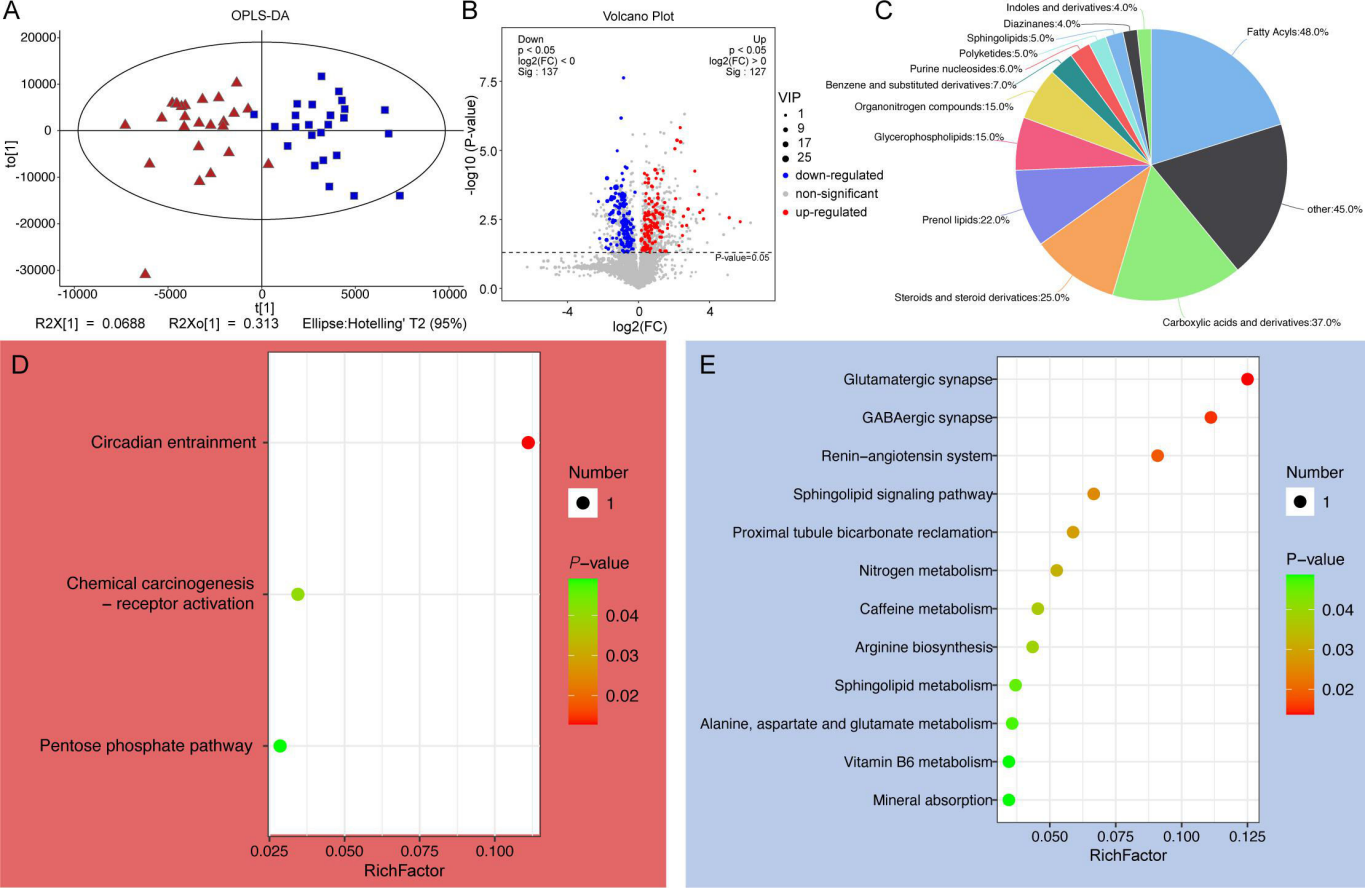
Metabolite composition and KEGG analysis. (**A**) Compositional patterns of metabolites in HDFI and HDFI groups. (**B**) Volcano plot showing differential metabolites between HDFI and HDFI groups. (**C**) Pie chart showing the percentage of the types of differential metabolites. KEGG analysis of upregulated (**D**) and downregulated differential metabolites (**E**).

Untargeted metabolomics analysis of seminal metabolomes from LDFI (*n* = 40) and HDFI (*n* = 47) patients identified significant alterations in metabolite profiles (Fig. 5A). A notable enrichment of 5,766 metabolites was found in HDFI samples (Fig. 5B), with a significant proportion being fatty acyls and carboxylic acids and derivatives (Fig. 5C). Further pathway analysis revealed that upregulated metabolites were significantly enriched in three KEGG pathways (Fig. 5D), whereas downregulated metabolites were associated with glutamatergic synapse, GABAergic synapse, and sphingolipid signaling pathways (Fig. 5E).

**Fig 6:**
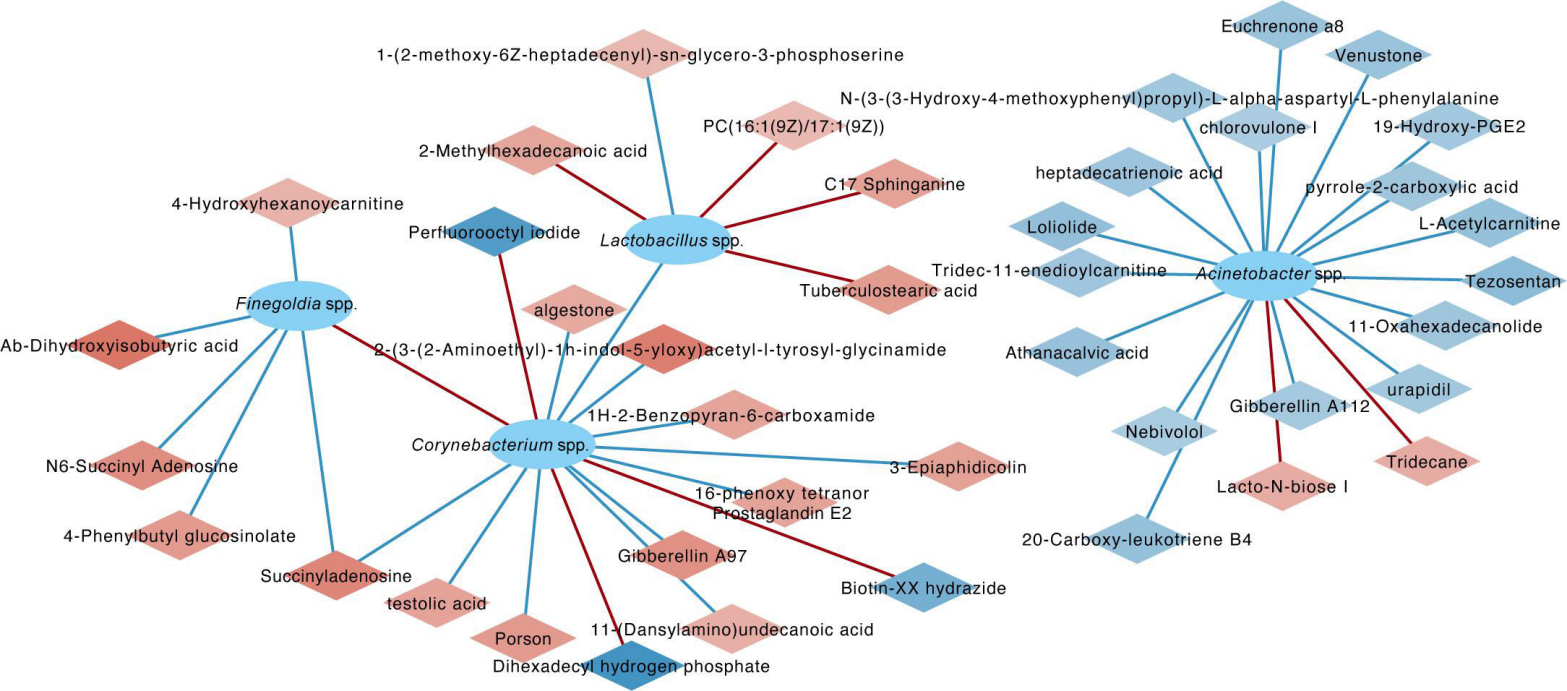
The co-occurrence network of differential metabolites and microbiota.

Integrated analysis of metabolites and microbiota demonstrated significant correlations, particularly between *Lactobacillus* spp. and specific unsaturated fatty acids, and negative correlations between *Corynebacterium* spp. and metabolites, including testolic acid, succinyladenosine, and 16-phenoxy tetranor prostaglandin E2, indicating a complex interaction between seminal microbiota and metabolite composition affecting sperm DFI (Fig. 6).

## DISCUSSION

Dysbiosis of the microbiome is closely related to semen quality. However, there was a paucity of research on its relationship with sperm DFI. Previous studies, such as the one conducted by Sergio et al., have leveraged full-length 16S rRNA gene sequencing to delineate the microbial characteristics of semen from infertile idiopathic patients. However, these investigations predominantly focused on idiopathic infertility, leaving the specific impact of seminal microbiota on DFI largely unexplored (13). Addressing this gap, our study expanded the patient cohort and integrated non-targeted metabolomics with 16S rRNA gene sequencing to elucidate the influence of seminal microbiota on sperm DFI, thereby revealing the detrimental impact of microbiota-derived metabolites on sperm DNA integrity.

Our comparative analysis identified distinct microbial compositions between HDFI and LDFI groups, with the HDFI group showing a prevalence of *Actinomycetaceae* and *Acinetobacter* spp., aligning with prior findings (13). Notably, our redundancy analysis pinpointed a correlation between *Lactobacillus* species levels and DFI, particularly highlighting variations in *Lactobacillus* species distribution between groups. The predominance of *L. iners* in the HDFI group, a species traditionally associated with the vaginal microbiome and indicative of dysbiosis due to its link with pro-inflammatory factors (24) and its ability to directly induce DNA damage in cancer cells (25), suggests a novel microbial marker for semen quality and DFI. DNA damage leads to an increase in intracellular phosphorylation of serine (26). *Lactobacillus iners* was negatively correlated with 1-(2-methoxy-6Z-heptadecenyl)-sn-glycero-3-phosphoserine, a compound that may be a product of DNA damage. Additionally, *Lactobacillus* spp. were negatively associated with *Corynebacterium* spp., and *L. iner*s, a species of *Lactobacillus* that lacks genes for the metabolism of amino acids and carbohydrates (27), may stimulate the growth of *Corynebacterium* spp. by producing unsaturated fatty acids (28–30), leading to a reduction in metabolites conducive to spermatogenesis.

With further clustering of the differentially distributed microbiota into KEGG Orthology prediction results in the HDFI group, we found a significantly higher activation level of the acetyl-CoA fermentation to butanoate II pathway compared with the LDFI group. The acetyl-CoA fermentation to butanoate II pathway involves a series of enzyme-catalyzed reactions that convert acetyl-CoA to butanoate (31). Butanoate salts are a class of compounds known as histone deacetylase inhibitors (HDIs). Histone deacetylases (HDACs) remove acetyl groups from lysine residues, resulting in condensed and transcriptionally silenced chromatin. HDIs block this action, potentially leading to hyperacetylation of histones (32), which may affect the conversion of sperm DNA histones to protamines, resulting in increased sperm DNA fragmentation. Furthermore, we observed an elevated level of purine nucleobase degradation I pathway in the HDFI group. The activity of the purine metabolic pathway correlates with DNA damage. Disruption in purine metabolism can lead to erroneous encoding of DNA (33), which may also be a potential cause of increased sperm DFI.

Our correlation analyses underscored *Acinetobacter* spp.’s close association with DFI levels, and we identified its correlation with antioxidant metabolites through correlation analysis. *Acinetobacter* sp. is an opportunistic pathogen, and it can produce DNA-damaging metabolites, such as angucyclines, anthracyclines, bleomycins, enediynes, mitomycins, and quinoxalines. These molecules are combined with DNA and cause either DNA distortion, alkylation, crosslinking, and/or oxidative damage (34). *Acinetobacter* spp. and other bacteria, including *Serratia* spp., *Pseudomonas* spp., and *Stenotrophomonas* spp., were identified in the genitourinary system and were related to reduction in sperm motility, lack in DNA integrity, destruction of mitochondrial functions and shape abnormalities of the sperms (35). El-Gendy and his colleagues performed subcutaneous injection of extracts obtained from *Acinetobacter* spp. to albino rats and found that sperm deformities percentage was statistically significantly increased (36). Therefore, we speculate that, in human semen, *Acinetobacter* spp. may deplete antioxidant metabolites and restrain the antioxidant capacity of sperm.

We recognize certain limitations in our study. For instance, our conclusions were based on 16S rRNA sequencing, which can only detect microbial communities statistically significant and not other organisms, such as fungi and viruses, and we did not conduct further experimental validation. Traditional microbial culture methods serve as the gold standard for clinical detection of microbiota and can also provide further analysis of drug resistance. However, some microbiota may not grow in common culture media or may have low abundance, making it difficult to validate the microbiota identified by next-generation sequencing using traditional microbial culture methods (37). Overall, our findings uncover a microbial perspective underlying sperm DNA integrity.

### Conclusions

This study not only provides a comprehensive overview of the metabolic interplay between microbiota and host in HDFI patient semen but also prompts further investigation into the microbiome’s role in semen quality. By combining non-targeted metabolomics and 16S rRNA gene sequencing, we offer insights into seminal microbiota’s potential adverse effects on sperm DNA, whether through antioxidant depletion or DNA-damage metabolite production. Future research is warranted to validate these findings and explore therapeutic interventions aimed at modulating the seminal microbiome to improve sperm quality and fertility outcomes.

## Data Availability

The raw sequence data reported in this paper have been deposited in the Genome Sequence Archive, which is developed and maintained by the Big Data Center at the Beijing Institute of Genomics, Chinese Academy of Sciences. The GSA system, accessible via http://bigd.big.ac.cn/gsa or http://gsa.big.ac.cn, adheres to the standards set by the International Nucleotide Sequence Database Collaboration (INSDC). Our data (GSA-Human: HRA007257) are publicly accessible at https://ngdc.cncb.ac.cn/gsa-human.

## SUPPLEMENTAL MATERIAL

Table S1 spectrum.00759-24-s0001.xlsx Result of cross-incubation experiments.

ASM does not own the copyrights to Supplemental Material that may be linked to, or accessed through, an article. The authors have granted ASM a non-exclusive, world-wide license to publish the Supplemental Material files. Please contact the corresponding author directly for reuse.
